# P-1005. Seroepidemiology of Cytomegalovirus, Rubella and Toxoplasma in Women of Childbearing Age in Korea

**DOI:** 10.1093/ofid/ofae631.1195

**Published:** 2025-01-29

**Authors:** Youngmin Cho, Hyejeong Moon, Eun Young Cho, Jaemin Park, Anna Lee, Hyunju Lee

**Affiliations:** Seoul National University Bundang Hospital, Seongnam-si, Kyonggi-do, Republic of Korea; Seoul National University Children’s Hospital, Seoul, Seoul-t'ukpyolsi, Republic of Korea; Chungnam National University Hospital, Daejeon , Not Applicable, Republic of Korea; Seoul National University Children’s Hospital, Seoul, Seoul-t'ukpyolsi, Republic of Korea; Seoul Medical Science Institute, Yongin, Kyonggi-do, Republic of Korea; Seoul National University Bundang Hospital, Seongnam-si, Kyonggi-do, Republic of Korea

## Abstract

**Background:**

This study aimed to assess the seroprevalence of Korean women of childbearing age to define the population susceptible to cytomegalovirus (CMV), rubella, and toxoplasmosis.

**Figure:**
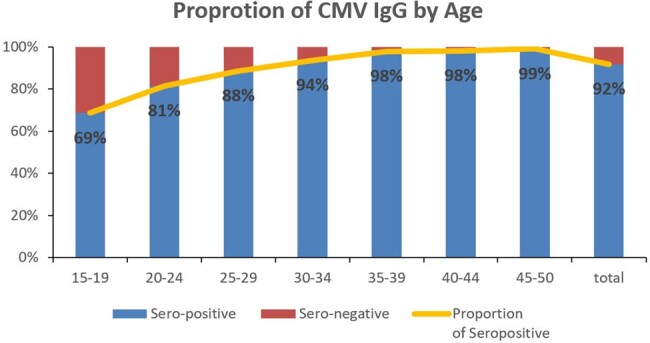

**Methods:**

The results of cytomegalovirus, rubella, and toxoplasma antibody tests performed by chemiluminescent microparticle immunoassay in women 15-50 years of age at Seoul Clinical Laboratories from 2012 to 2022 were analyzed based on age, year, and region.

**Figure:**
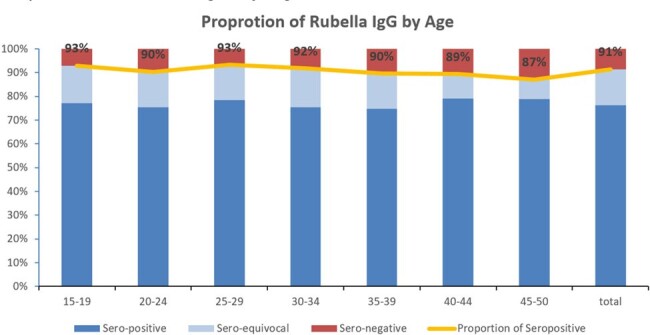

**Results:**

The average positive seroprevalence rate was 92% (256/3,114) for CMV, 91% (405,207/442,998) for rubella, and 5% (819/15,712) for toxoplasma. In the age-group analysis, seroprevalence of CMV increased from 69% in women 15-19 years to ≥98% in 35-50 years of age, whereas seroprevalence of rubella was higher in women 15-39 years (90-93%) than in women 40-50 years (80-89%) of age. There was no change between age groups in seroprevalence of toxoplasma.

**Figure:**
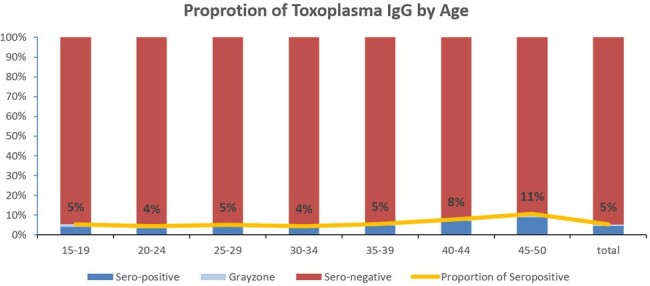

**Conclusion:**

During the past 10 years, there was no significant difference of seroprevalence for CMV, rubella, and toxoplasma according to year or region age in reproductive women in Korea. To prevent and manage congenital infections, monitoring for the changes in seroprevalence of women with childbearing age should be continued.

**Disclosures:**

**All Authors**: No reported disclosures

